# Protocol for preparation of heterogeneous biological samples for 3D electron microscopy: a case study for insects

**DOI:** 10.1038/s41598-021-83936-0

**Published:** 2021-02-25

**Authors:** Alexey A. Polilov, Anastasia A. Makarova, Song Pang, C. Shan Xu, Harald Hess

**Affiliations:** 1 Department of Entomology, Faculty of Biology, Moscow State University, Moscow, Russia; 2 Janelia Research Campus of the Howard Hughes Medical Institute, Ashburn, USA

**Keywords:** Imaging, 3-D reconstruction, Zoology, Entomology, Microscopy, Scanning electron microscopy, Transmission electron microscopy

## Abstract

Modern morphological and structural studies are coming to a new level by incorporating the latest methods of three-dimensional electron microscopy (3D-EM). One of the key problems for the wide usage of these methods is posed by difficulties with sample preparation, since the methods work poorly with heterogeneous (consisting of tissues different in structure and in chemical composition) samples and require expensive equipment and usually much time. We have developed a simple protocol allows preparing heterogeneous biological samples suitable for 3D-EM in a laboratory that has a standard supply of equipment and reagents for electron microscopy. This protocol, combined with focused ion-beam scanning electron microscopy, makes it possible to study 3D ultrastructure of complex biological samples, e.g., whole insect heads, over their entire volume at the cellular and subcellular levels. The protocol provides new opportunities for many areas of study, including connectomics.

## Introduction

In recent years, methods for 3D study of the ultrastructural organization of organisms have been actively developed. Until recently, the only way to study spatial organization at the cellular and subcellular levels at a high definition was to examine series of ultrathin sections using a transmission electron microscope (TEM), now a number of new methods and devices have been developed^1–3^, such as the automated tape-collecting ultramicrotome (ATUM^4^), scanning electron microscope with integrated microtome (SBF-SEM^5^), focused ion-beam scanning electron microscope (FIB-SEM^6–8^), gas cluster ion beam scanning electron microscope (GCIB-SEM^9^), and others. Modern 3D electron microscopes make it possible to quickly and efficiently obtain information on the structure of rather large samples at the subcellular level, which makes a considerable contribution to understanding the structure and ultrastructure of cells and tissues. For examining samples of insects and other organisms that have hard integuments, these methods can be insufficiently useful, since serial cutting of these samples is extremely difficult due to the chipping cuticle, which deforms the sections.

Classical methods for preparing samples for electron microscopy do not provide sufficient contrast of samples, because they were designed for using additional staining of sections^10–12^, which is impossible with many methods of 3D-EM. The widely used sample preparation methods for FIB-SEM and SBF-SEM work well only on pieces of homogeneous tissue^13,14^. Currently, protocols are being actively developed that will enable the study of large tissue samples^15^, such as whole fruit fly brains^16–18^, zebrafish brains^19^, or even mouse brains^20,21^ but these protocols cannot be used for the en block staining of whole insect head, since they do not provide uniform quality for a heterogeneous sample (consisting of dissimilar tissues dissimilar in structure and composition). The success of specimen fixation and contrasting is largely determined by the rate of penetration of the reagents into the sample^11,22^, which is considerably slowed down in heterogeneous samples with the presence of a poorly permeable integument or of tissues with a high lipid content. Such problems with sample preparation are noted for insects^23,24^ and other arthropods ^25^, other invertebrates^25–28^ and vertebrates^29^, as well as plants ^30^.

The goal of this study is to develop a protocol for the preparation of heterogeneous biological samples for 3D-EM. The 3D-EM methods in connectomics are most in demand; our attention was therefore focused on the brains of the sampled insects.

## Results and discussion

**Figure 1:**
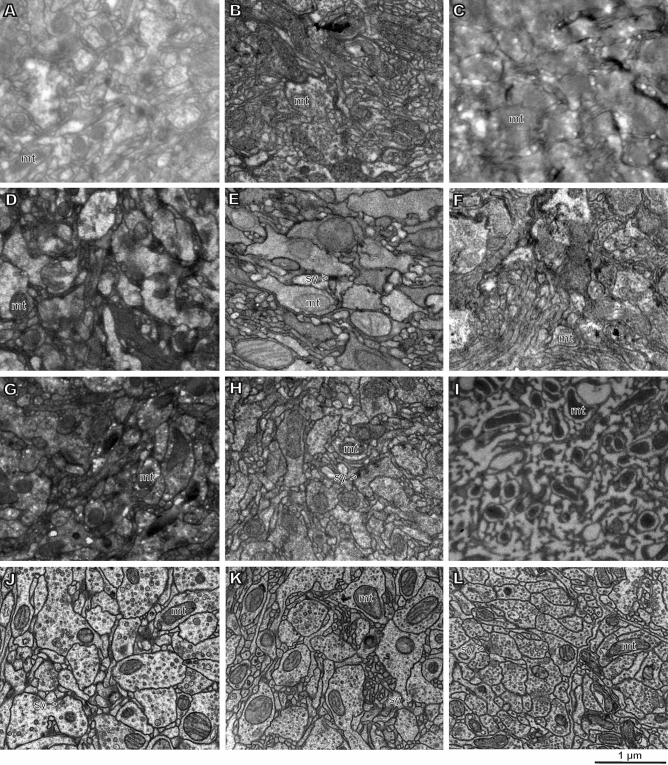
Comparison of the results of various protocols for preparation of whole heads of parasitoid wasp *Megaphragma amalphitanum* (TEM, areas of neuropil from middle regions of the brain). (**A**) Classical fixation with GA and staining with OsO_4_^10^; (**B**) OTO^34^; (**C**) GA + PFA, RO and TA^13^; (**D**) GA + PFA, OTO, UA, PbAsp^14^; (**E**) GA + PFA, ROTO, UA, PbAsp^35^; (**F**) GA, BROPA^21^; (**G**) GA + PFA, OsO_4_, FeCN, TCH, OsO_4_, UA, PbAsp ^15^; (**H**) GA + PFA, PLT^16^; (**I**) HPF and ASF^16^; (**J**) our protocol; (**K**) our protocol accelerated; (**L**) our protocol MW. mt, mitochondrion; sy, synapse.

**Figure 2:**
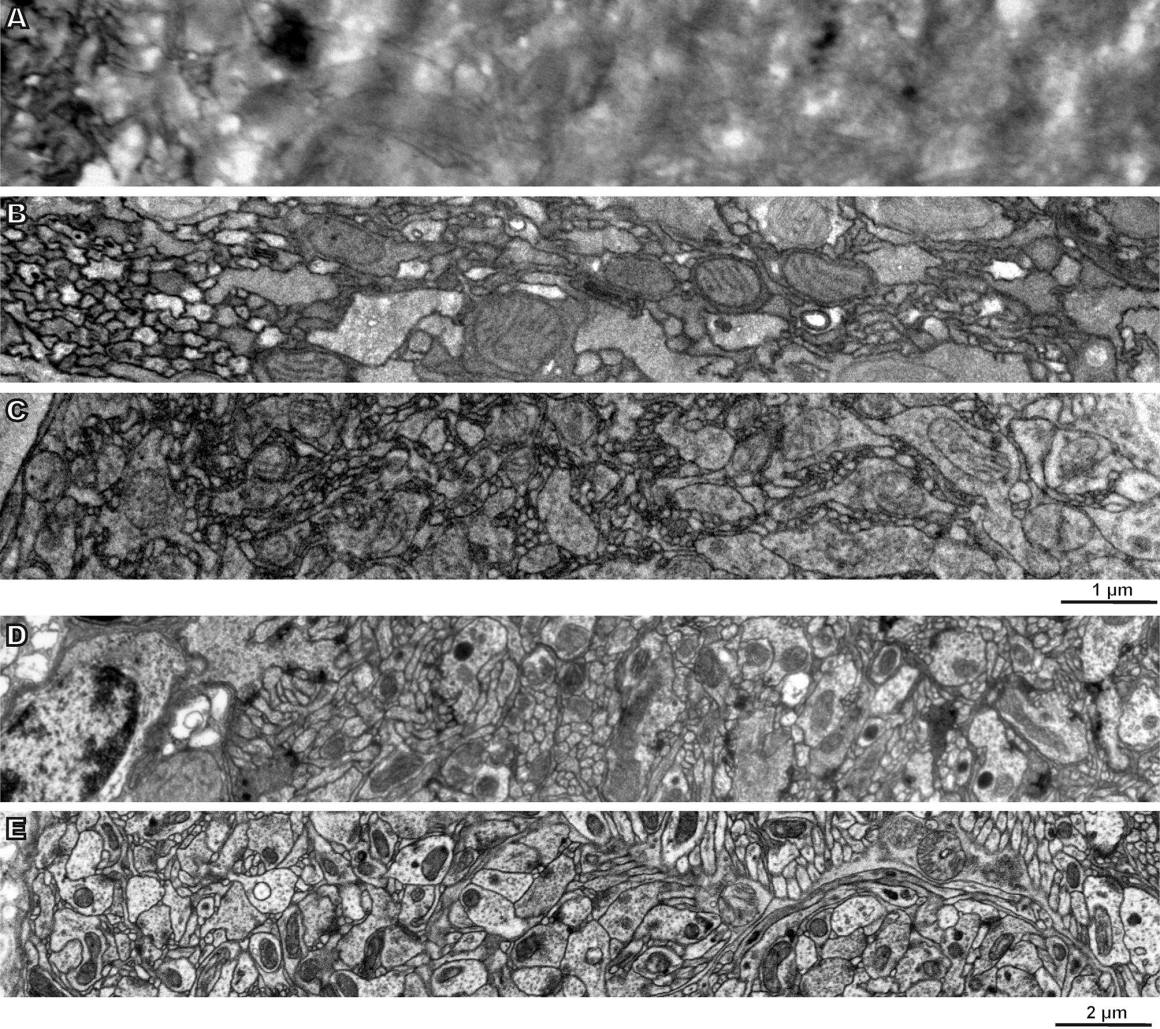
Comparison of the homogeneity of contrasting for samples of the brain of the parasitoid wasp *Megaphragma amalphitanum* from peripheral regions (left) to central regions (right) in samples prepared according to different protocols (TEM). (**A**) RO and TA^13^; (**B**) ROTO, UA, PbAsp^35^; (**C**) Permanganate^36^; (**D**) PLT^16^; (**E**) our protocol.

In the search for the optimal protocol, we have tested all currently used protocols for preparing samples for 3D-EM, but all of them do not provide acceptable results for studying whole insect heads (Figs. 1, 2, S1, S2). We have also tested over 300 protocols combining various options for all stages of sample preparation (Table S1).

As a result, we have developed a protocol based on simultaneous fixation with glutaraldehyde (GA) and osmium (OsO_4_)^31^, sequential osmification and treatment of samples with potassium ferrocyanide (FeCN)^15^, staining of samples with uranyl acetate (UA) with heating to 50 °C^32^, staining with lead aspartate (PbAsp)^33^. Other important components of our protocol include prolonged osmification, absence of moderators and of subsequent second osmification, prolonged washing between stages, and great duration of exposure during dehydration and embedding.

Fixation is one of the most critical stages in the preparation of heterogeneous samples; this problem is especially acute for samples of insects, since their cuticle is impermeable or weakly permeable to most of the fixatives used, and their fat bodies, rich in lipids, also cause difficulties. Standard fixation, GA or GA + formaldehyde (PFA), does not allow attaining sufficient fixation quality (Fig. 1A,C–H). The use of additional agents (picric acid, tannic acid, etc.) in combination with aldehyde fixation improves the quality of fixation, but increases the uneven penetration of fixatives and contrast agents or reduces the contrast between cell structures and cytoplasm. Primary fixation with an osmium solution does not allow obtaining preparations with well-preserved membranes (Fig. 1B), and samples fixed with reduced osmium solution (RO, mixture of OsO4 and FeCN) have a low contrast and are additionally spoiled by precipitation of metals. Permanganate fixation allows obtaining very contrasting images of membranes, but other components of cells are destroyed, and the fixation is uneven. High-pressure freezing with subsequent automatic freeze substitution (HPF + AFS) does not provide the needed quality and uniformity of fixation (Fig. 1I), apparently due to the fact that the cuticle can play the role of a thermal barrier. The best quality of the samples was obtained using simultaneous fixations with 1% GA + 1% OsO_4_ (Fig. 1J–L). For the other tested variants of fixation and subsequent stages, see Table S1.

The second critical stage is the staining of the samples. The standard single osmification is not enough to obtain the desired level of contrast (Fig. 1A). Staining with RO provides for good preservation of the ultrastructure and for high contrast, but it is accompanied by precipitation of metals in the tissues. Staining with permanganate allows obtaining very contrasting membranes, but the other components of the cells are completely destroyed, and the contrast is uneven throughout the depth of the sample (Fig. 2C). Multiple osmification (OTO^34^, ROTO^35^, ROTAO^13^, BROPA^21^, etc.) in combination with moderators [thiocarbohydrazide (TCH), tannic acid (TA), pyrogallol, etc.] makes it possible to obtain contrasting samples, but the contrast level is uneven throughout the depth of the sample (Fig. 2A,B) and precipitation takes place in tissues (Fig. 1C–G). Staining after progressive lowering of temperature (PLT)^16^ together with standard fixations does not provide for even fixation quality and contrast of the sample (Fig. 1H). Testing various staining methods has shown that the optimal method is multistep staining with sequential treatment of the samples with OsO_4_, FeCN, UA, PbAsp (Fig. 1J–L). Heating to 50 °C at the last two stages allows considerably increasing the contrast.

Comparison of different dehydration variants has shown that the optimal variant is to use the ethanol series (EtOH 30%, 50%, 70%, 95%, 100%) at 4 °C, followed by final dehydration in acetone (Ac) or propylene oxide (PO). Dehydration only in Ac or PO leads to the deformation of tissues. Due to the presence of the cuticle, sufficiently long exposure durations are required at all stages of dehydration (at least 30–60 min at each stage).

A comparison of the results of scanning samples concluded in Epon, Durcupan, Araldite, Spurr, Hard-Plus resin. We have shown that the most contrasting and detailed picture is obtained using Epon 812. On the other hand, Durcupan and Hard-Plus resin give most moderate of artifacts when examining the samples using FIB-SEM, which makes it possible to scan larger samples.

**Figure 3:**
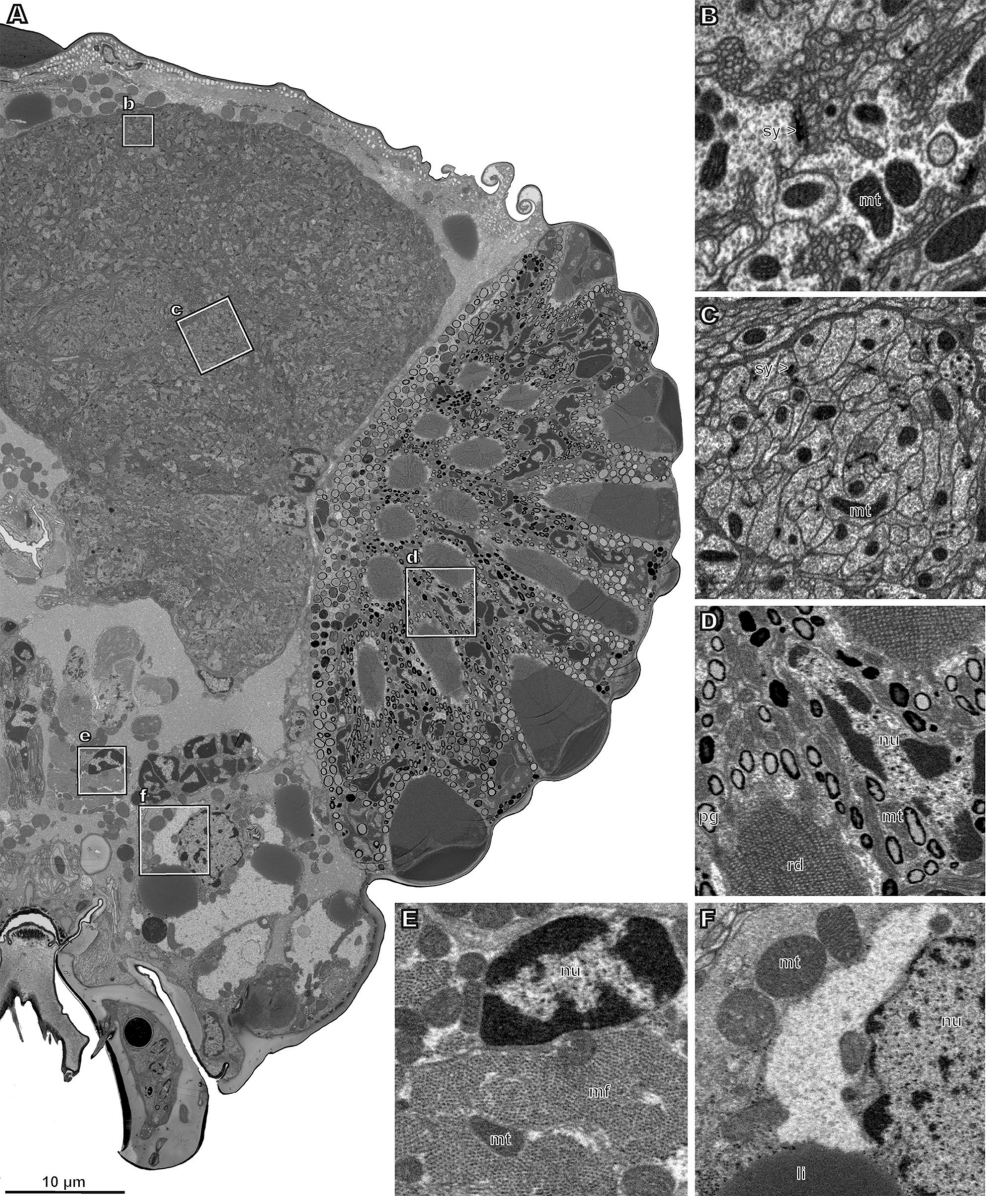
Half of one cross section of the complete 3D-EM (FIB-SEM) series of the whole head of parasitoid wasp *Megaphragma amalphitanum* (**A**) and close-up of its fragments (**B–F**). (**B**) neuropil of a peripheral region of the brain; (**C**) neuropil of the central region of the brain; (**D**) central area of compound eye; (**E**) muscle; (**F**) fat body. Li, lipid inclusion; mf, muscular fiber; mt, mitochondrion; nu, nucleus; pg, pigment granule; rd, rhabdom; sy, synapse. Sample prepared according to our protocol. For sections of the same stack in other planes, see Figs S1, S2.

Samples prepared according to our protocol combine a high degree of preservation of the anatomy and ultrastructure with a contrast sufficient for studying the samples using FIB-SEM, SBF-SEM, and TEM without staining the sections (Figs. 1J, 2E, 3). The variants of the protocol at different stages of optimizing it have already been used to study the ultrastructure of the eyes of featherwing beetles (Coleoptera: Ptiliidae)^24^, the connectome of the optic lobes of *Megaphragma* wasps (Chua et al., in prep.), and the ultrastructure of the sensory organs of *Megaphragma* wasps (Diakova et al., in prep; Makarova et al., in prep.).

Many modern methods of sample preparation require complex and expensive equipment (High-pressure freezer, Microwave processor, Automatic freeze substitution system). The protocol we have developed does not require anything but standard equipment available in most laboratories working with EM (Supplement).

If necessary, this time can be reduced to 3 days by replacing most stages at 4 °C with shorter ones at room temperature (RT) and polymerization of the embedding medium at a higher temperature (Fig. 1K). But an increase in temperature leads to tissue shrinks, which in combination with the strong cuticle of the head can result in the rupture of tissues attached to it. Another option to reduce sample preparation time is to use microwave radiation to accelerate the processes (Fig. 1L), but in this case the level and evenness of contrast are not always good. For different samples, depending on their sizes and permeability, it may be necessary to optimize our protocol, mainly by changing the selection of the optimal concentration of fixatives and fixation duration at stages I, III, and V of sample preparation, and by the selection of the optimal molarity of the buffer used. For small and/or highly permeable samples, the concentration of the fixatives at stages I and V and the duration of stages I, III and V can be halved, as well as the duration of washing the samples after these stages and the duration of dehydration. For large and/or poorly permeable samples, it is possible to increase the fixation time at stage I to 85 min and at stage II by a factor of 2–3. Recently proposed modifications of PLT^18^ could possibly allow to complement our protocol and further improve the contrast of the samples.

The choice of the microscope is also instrumental for successfully solving the problems of 3D-EM. Working with SBF-SEM has its advantages and disadvantages. The advantages include the high speed of this work and the opportunity to obtain images of large samples (their size limited by the size of the diamond knife and the required Z step). But the disadvantages make this method useless for many objectives. The main disadvantage is the fact that obtaining thin sections from samples that include the cuticle or other hard components often leads to chipping of the cuticle and to deformation of the block surface, which distorts the resulting image. But for soft tissues SBF-SEM makes it possible to obtain high-quality images of samples prepared according to our protocol (Fig. S3). Using FIB-SEM solves problems that arise when working with SBF-SEM, but ion etching also has some limitations, mainly those concerning the etching depth, which is limited by the ability to focus the ion beam. Different kinds of ion scanning microscopes can produce data of different quality. A modified FIB-SEM used in the laboratory of Harald Hess (Janelia)^7,8^, in which the ion column and electron column are oriented at 90 degrees to each other and several multilevel control systems for the main components of the system are introduced. This microscope allows obtaining high-resolution (4 × 4 × 4 nm along X × Y × Z) and high-contrast images for samples of large volumes (the system is able to work stably without stopping for several months and makes it possible to study samples up to 100 µm thick)^7,8^. FIB-SEM settings require rather many preliminary experiments for each sample type, since the choice of the ion beam current, electron gun voltage, scanning frequency, and many other parameters depend on the type of the sample, its size, the staining variant, and the embedding medium.

## Conclusions

As a result of testing the principal protocols used to prepare samples for electron microscopy and different variants of combinations of all their stages, we have developed a simple protocol for sample preparation of heterogeneous biological samples for 3D-EM. This protocol can be useful for studying the ultrastructure of various organisms using FIB-SEM and SBF-SEM and for facilitating work with TEM, since it does not require staining of sections. The protocol has been successfully tested on various insects; it allows making preparations of whole heads or even whole bodies, giving new opportunities for large-scale studies of the ultrastructure of organisms at the cellular and subcellular levels. It is especially useful for connectomics, helping to study not only the brain at the cellular level but also the ultrastructure of receptors and their projections into the brain and the effector pathways that exit the brain. All this together suggests that the protocol will be in demand for solving various problems by researchers working in a broad range of areas.

## Materials and methods

### Studied species

The main model used in this study is the parasitoid wasp *Megaphragma amalphitanum* Viggiani, 1997 reared in the laboratory from eggs of *Heliothrips haemorrhoidalis* (Bouché, 1833). The principal variants of sample preparation were tested also on featherwing beetles *Nephanes titan* (Newman, 1834) collected at the Zvenigorod Biological Station, Lomonosov Moscow State University, and on the fruit fly *Drosophila melanogaster* Meigen, 1830 from a culture maintained at the Department of Genetics, Faculty of Biology, Lomonosov Moscow State University.

### Protocol

All our experiments were performed on whole heads, which were separated from the body in a fixative on glass slides with cavities and immediately transferred into fresh fixative. Fixation, staining, dehydration, and infiltration were performed in plastic tubes; the volume of all liquids was at least 1000 times as great as that of the sample; the process solutions were changed automatically or with a disposable dropper without transferring the sample from one tube to another. For the chemicals, supplies, and equipment, see Supplement.

I. Immediately after dissection in fixative 1, the samples were transferred to fresh fixative 1 and kept for 45 min at 4 °C (Toxic). Fixative 1, 1% glutaraldehyde (GA) and 1% osmium tetroxide (OsO_4_) in 0.1 M cacodylate buffer, pH 7.2 (CB). (Critical: prepare the fixative immediately prior to fixation, monitoring the temperature at all stages of fixation; if the samples are poorly wettable and adhere to the surface film of the liquid, it is necessary to make them sink and make sure that they do not float at this stage or at any of the subsequent stages)
II. Washing samples in 0.1 M CB, two changes with a total duration of 20 min at 4 °C (Toxic)
III. Fixation with fixative 2 (2% GA in 0.1 M CB) for 2 h at 4 °C (Toxic)
IV. Washing the samples in 0.1 M CB, four changes with a total duration of 2.5 h at 4 °C (Toxic)
V. Postfixation and staining with a buffer solution of osmium tetroxide (2% OsO_4_ in 0.1 M CB) for 12 to 20 h at 4 °C (Toxic)
VI. Treatment with buffer solution with potassium ferrocyanide (1% FeCN in 0.1 M CB) for 2 h at 4 °C (Toxic)
VII. Washing the samples with double distilled water (ddH_2_O), four changes with a total duration of 2.5 h at 4 °C
VIII. Staining with an aqueous solution of uranyl acetate (UA) with heating to 60 °C (1% UA in ddH_2_O for 8–12 h at 4 °C, then in the same solution for 2 h at 50 °C in a constant temperature oven). (Critical: use only fresh ddH_2_O) (Toxic)
IX. Washing the samples with ddH_2_O, four changes with a total duration of 2 h at RT
X. Staining with lead aspartate according to Walton (PbAsp, 0.66% lead nitrate in 0.03 M aspartic acid, pH adjusted to 5.5 with 1 M KOH) for 2 h at 50 °C (Toxic)
XI. Washing the samples with ddH_2_O, four changes with a total duration of 2.5 h at RT
XII. Dehydration in ethanol of increasing concentrations (EtOH) and acetone (Ac)
1. EtOH 30% for 30 min at 4 °C
2. EtOH 50% for 30 min at 4 °C
3. EtOH 70% for 60 min at 4 °C
4. EtOH 95% for 60 min at 4 °C
5. EtOH 100% for min at 4 °C. (Critical: dry EtOH)
6. EtOH 100% for 30 min at RT. (Critical: dry EtOH)
7. EtOH 100% + Ac for 30 min at RT. (Critical: dry EtOH and Ac)
8. Ac for 30 min at RT. (Critical: dry Ac)
9. Ac for 30 min at RT. (Critical: dry Ac)
XIII. Placing in embedding medium (Epon 812)
1. Mixture of Epon and acetone (1: 2) for 2 h at RT in rotator at 1 rpm. (Critical: dry Ac) (Toxic)
2. Mixture of Epon and acetone (1: 1) for 24 h at RT in rotator at 1 rpm. (Critical: dry Ac) (Toxic)
3. Epon for 2 h at RT in rotator at 1 rpm (Toxic)
4. Epon for 5 h at RT in rotator at 1 rpm (Toxic)
XIV. Polymerization of Epon for 48 h at 60 °C in silicon embedding molds. (Toxic)

### Protocol variants

*Accelerated sample preparation.* Sequence and the contents of the operations are the same as in the original protocol, but stages II–VII at RT, stage XIV at 95 °C, duration of stages III, IV, VI, VII 1 h, duration of stage V 4 h, and duration of stage XIV 2 h.

*Using a microwave processor.* The sequence and contents of the operations is the same as in the original protocol, but stages I–VII in a microwave processor in an ice bath at a power of 200 W according to the following On (Off) scheme for microwaves:

- I—0.5 (0.5) 0.5 (0.5) 0.5 (1) 0.5 (1) 0.5 (10) 1 (10) 1 (10)
- II—0.5 (5)
- III—0.5 (0.5) 0.5 (0.5) 1 (1) 1 (20) 1 (30)
- IV—0 0.5 (5), change of 0.1 M CB, 1 (30), change of 0.1 M CB, 1 (30)
- V—0.5 (1) 0.5 (1) 0.5 (1) 1 (20) 1 (30)
- VI—0.5 (1) 0.5 (1) 0.5 (1) 1 (10) 1 (20)
- VII—0.5 (5), change of ddH20, 1 (30), change of ddH20, 1 (30)
- XIV—2 h at 95 °C in microwave processor or constant temperature oven.

### Microscopy

Assessment of the preservation of the general anatomy was performed using a series of histological sections made using a Leica RM 2255 microtome and studied under an Olympus BX43 microscope. Initial assessment of the quality of the ultrastructure of transmission electron microscope samples (Jeol JEM-1011 and JEM-1400, Lomonosov Moscow State University) was performed on sections of 40–50 nm made using a Leica UC6 ultramicrotome; these samples at the early stages of the choice of fixatives were standard stained with lead citrate and UA and at subsequent stages, including en block staining, were studied without additional staining of sections. Quality samples were then tested at FIB-SEM (FEI Quanta FEG, Moscow State University, or FEI Helios, Kurchatov Institute), and the best samples were sent to the Janelia Research Campus (United States) for scanning on a modified FIB-SEM (Zeiss Merlin + Capella) or to the FEI Research Center (United States) for scanning on a SBF-SEM (FEI Teneo). Before scanning, sample quality was evaluated using an X-ray micro-CT (Xradia Versa 3D XRM-510). The large-scale imaging of the *Megaphragma* was done with a Zeiss Merlin scanning electron microscope that has a Zeiss Capella focused ion beam column mounted on at 90 degrees and controlled by custom software^7,8^. To image an entire head we used 2 MHz pixel rates with a 2nA primary electron beam with final voxels that were sampling at 8 × 8 nm in x and y and milled with effective 8 nm increments. To image such large volumes, (> 100 micron size) required continuous stable image acquisition for about a month.

## Supplementary Information

Supplementary Information
