# Supplementary material for: Protocol for preparation of heterogeneous biological samples for 3D electron microscopy: a case study for insects
Source: Sci Rep. 2021 Feb 25;11:4717. doi: 10.1038/s41598-021-83936-0 (PMC7907262; doi:10.1038/s41598-021-83936-0)
Supplement: Supplementary file 1 — Supplementary Information [file 41598_2021_83936_MOESM1_ESM.pdf]

Protocol for preparation of heterogeneous biological samples for 3D electron microscopy: A case study for insects

Alexey A. Polilov [1*] Anastasia A. Makarova[1], Song Pang[2], C. Shan Xu[2], Harald Hess[2]

[1] Department of Entomology, Faculty of Biology, Moscow State University, Moscow, Russia
[2] Janelia Research Campus of the Howard Hughes Medical Institute, United States

* Correspondence to polilov@gmail.com

Supplementary materials:

Table S1. Principal variants of the main stages of sample preparation that have been tested during our work on developing the protocol

Figure S1. Sagittal section of complete 3D-EM (FIB-SEM) series of the whole head of the parasitoid wasp *Megaphragma amalphitanum* (same stack as in Fig. 3, S2).

Figure S2. Frontal section of complete 3D-EM (FIB-SEM) series of the whole head of the parasitoid wasp *Megaphragma amalphitanum* (same stack as in Fig. 3, S1).

Figure S3. Examples of SBF-SEM images of central portions of the neuropil of the brain of the parasitoid wasp *Megaphragma amalphitanum*.

List of chemicals

List of equipment and supplies

Table S1. Principal variants of the main stages of sample preparation that have been tested during our work on developing the protocol

	**Principal variants**	**Conditions**
**Fixation**	Chemical fixation using solution of:<br>1. Glutaraldehyde (GA)[1].<br>2. Glutaraldehyde and formaldehyde (GA+PFA)[1,2].<br>3. Glutaraldehyde, formaldehyde and таниновой кислоты (GA+PFA+TA)[1].<br>4. Glutaraldehyde and hydrogen peroxide (GA+H_2O_2)[3].<br>5. Glutaraldehyde, formaldehyde and ruthenium red (GA+PFA+RR)[1].<br>6. Glutaraldehyde, formaldehyde and picric acid (GA+PFA+PA)[1,4]<br>7. Osmium tetroxide (OsO_4)[1].<br>8. Osmium tetroxide and potassium ferrocyanide (OsO_4+FeCN)[5]<br>9. Glutaraldehyde and osmium tetroxide (GA+ OsO_4)[6,7].<br>10. Permanganate ($KMnO_4$ and $NaMnO_4$)[8].	• Various buffers (phosphate buffer, cacodylate buffer, without buffer, 0.05 M–0.2 M, pH 7.0–7.5).<br>• Fixation conditions (temperature: 0 °C, 4 °C, room temperature (RT); duration: 0.5–24 h)[9].<br>• Fixative concentration (0.1–4.0%).<br>• Fixation modifiers (Triton, DMSO, sucrose, calcium chloride).<br>• Fixation in microwave processor[10,11].
	High-pressure freezing with subsequent automatic freeze substitution (HPF + AFS) [12] [13] [14] [15]	• Cryoprotectant testing (BSA, glycerol, sucrose, hexadecene).<br>• Variants of ASF protocols (duration, temperature regime, and composition of mixture for AFS)[12-17].
**Staining**	I. Variants of contrast agents:<br>1. Osmium teroxide (OsO_4).<br>2. Osmium tetroxide and potassium ferrocyanide (OsO_4+FeCN).<br>3. Potassium or sodium permanganate ($KMnO_4$ and $NaMnO_4$)[1].<br>4. Osmium tetroxide solution and formamide[18].<br>5. Periodic acid.<br>6. Uranyl acetate (UA)[19].<br>7. Lead aspartate (PbAsp)[20].<br>8. Lead citrate and copper sulphate[21].<br><br>II. Staining methods<br>1. Single staining.<br>2. Multiple staining using alternating contrast agents (OsO_4, OsO_4+FeCN) and moderators (thiocarbohydrazide, tannic acid, pyrogallol, etc.)[18,21-27].<br>3. Multistep staining using various contrast agents (additional staining with uranyl acetate (UA), lead aspartate (PbAsp), periodic acid, lead citrate and copper sulphate)[21,24-26,28,29].<br>4. Progressive lowering of temperature (PLT) and low temperature staining[14,30,31].	• Concentrations, composition of solutions (aqueous, buffer, alcoholic).<br>• Temperature (4 °C, RT, 60 °C).<br>• Duration of exposure (30 min to 48 h).<br>• Contrasting in microwave processor[10].
**Dehydration and embedding**	1. Selection of dehydration agent (ethanol (EtOH), acetone (Ac), propylene oxide (PO), and their compbinations)<br>2. PLT[14,30,31].<br>3. Testing of principal embedding media for electron microscopy and of protocols for embedding samples in them (Epon, Durcupan, Araldite, Spurr, Hard-Plus resin)[32].	• Concentrations of solutions<br>• Duration of exposure[33].<br>• Temperature of dehydration (0 °C, 4 °C, RT, PLT).<br>• Using microwave processor[10].

References for Table S1

1. Hayat, M. A. *Fixation for electron microscopy*. (Academic Press, 1981).
2. Karnovsky, J. M. A formaldehyde-glutaraldchydc fixative of high osmolality for use in electron microscopy. *J. Cell Biol.* **27**, 137-139 (1965).
3. Peracchia, C. & Mittler, B. S. Fixation by Means of Glutaraldehyde-Hydrogen Peroxide Reaction Products. *J. Cell Biol.* **53**, 234-238 (1972).
4. Somogyi, P. & Takagi, H. A note on the use of picric acid-paraformaldehyde-glutaraldehyde fixative for correlated light and electron microscopic immunocytochemistry. *Neuroscience* **7**, 1779-1783 (1982).
5. Goldfischer, S., Kress, Y., Coltoff-Schiller, B. & Berman, J. Primary fixation in osmium-potassium ferrocyanide: the staining of glycogen, glycoproteins, elastin, an intranuclear reticular structure, and intercisternal trabeculae. *J. Histochem. Cytochem.* **29**, 1105-1111 (1981).
6. Eisenman, E. A. & Alfert, M. A new fixation procedure for preserving the ultrastructure of marine invertebrate tissues. *J. Microsc.* **125**, 117-120 (1982).
7. Hall, D. H., Hartwieg, E. & Nguyen, K. C. Q. in *Methods Cell Biol.* Vol. Volume 107 (eds H. Rothman Joel & Singson Andrew) 93-149 (Academic Press, 2012).
8. Luft, J. H. Permanganate; a new fixative for electron microscopy. *J. Biophys. Biochem. Cytol.* **2**, 799-802 (1956).
9. Maunsbach, A. B. & Afzelius, B. A. *Biomedical Electron Microscopy*. (Academic Press, 1999).
10. Giberson, R. T., Demaree, R. S. J. & (Eds.). *Microwave Techniques and Protocols*. (Humana Press, 2001).
11. Fischer, S., Lu, Z. & Meinertzhagen, I. A. From two to three dimensions: The importance of the third dimension for evaluating the limits to neuronal miniaturization in insects. *J. Comp. Neurol.* **526**, 653-662 (2017).
12. Walther, P. & Ziegler, A. Freeze substitution of high-pressure frozen samples: the visibility of biological membranes is improved when the substitution medium contains water. *J. Microsc.* **208**, 3-10 (2002).
13. Sosinsky, G. E. *et al.* The combination of chemical fixation procedures with high pressure freezing and freeze substitution preserves highly labile tissue ultrastructure for electron tomography applications. *J. Struct. Biol* **161**, 359-371 (2008).
14. Hayworth, K. J. *et al.* Ultrastructurally smooth thick partitioning and volume stitching for large-scale connectomics. *Nat. Methods* **12**, 319-322 (2015).
15. Takemura, S. Y. *et al.* A visual motion detection circuit suggested by *Drosophila* connectomics. *Nature* **500**, 175-181 (2013).
16. Jiménez, N. *et al.* Tannic acid-mediated osmium impregnation after freeze-substitution: A strategy to enhance membrane contrast for electron tomography. *J. Struct. Biol* **166**, 103-106 (2009).
17. Giddings, T. H. Freeze-substitution protocols for improved visualization of membranes in high-pressure frozen samples. *J. Microsc.* **212**, 53-61 (2003).
18. Mikula, S. & Denk, W. High-resolution whole-brain staining for electron microscopic circuit reconstruction. *Nat. Methods* **12**, 541-546 (2015).
19. Locke, M., Krishnan, N. & McMahon, J. T. A routine method for obtaining high contrast without staining sections. *J. Cell Biol.* **50**, 540-544 (1971).
20. Walton, J. Lead aspartate, an en bloc contrast stain particularly useful for ultrastructural enzymology. *J. Histochem. Cytochem.* **27**, 1337-1342 (1979).
21. Tapia, J. C. *et al.* High-contrast en bloc staining of neuronal tissue for field emission scanning electron microscopy. *Nat. Protoc.* **7**, 193-206 (2012).
22. Willingham, M. C. & Rutherford, A. V. The use of osmium-thiocarbohydrazide-osmium (OTO) and ferrocyanide-reduced osmium methods to enhance membrane contrast and preservation in cultured cells. *J. Histochem. Cytochem.* **32**, 455-460 (1984).
23. Seligman, A. M., Wasserkrug, H. L. & Hanker, J. S. A new staining method (OTO) for enhancing contrast of lipid-containing membranes and droplets in osmium tetroxide-fixed tissue with osmiophilic thiocarbohydrazide (TCH). *J. Cell Biol.* **30**, 424-432 (1966).
24. Bushby, A. J. *et al.* Imaging three-dimensional tissue architectures by focused ion beam scanning electron microscopy. *Nat. Protoc.* **6**, 845-858 (2011).
25. Hua, Y., Laserstein, P. & Helmstaedter, M. Large-volume en-bloc staining for electron microscopy-based connectomics. *Nat. Commun.* **6**, 7923 (2015).
26. Mikula, S., Binding, J. & Denk, W. Staining and embedding the whole mouse brain for electron microscopy. *Nat. Methods* **9**, 1198-1201 (2012).
27. Genoud, C., Titze, B., Graff-Meyer, A. & Friedrich, R. W. Fast Homogeneous En Bloc Staining of Large Tissue Samples for Volume Electron Microscopy. *Front. Neuroanat.* **12**, 76 (2018).
28. Briggman, K. L. & Bock, D. D. Volume electron microscopy for neuronal circuit reconstruction. *Curr. Opin. Neurobiol.* **22**, 154-161 (2012).
29. Holcomb, P. S. *et al.* Synaptic inputs compete during rapid formation of the calyx of Held: a new model system for neural development. *J. Neurosci.* **33**, 12954-12969 (2013).

30. Lu, Z. *et al. En bloc* preparation of *Drosophila* brains enables high-throughput FIB-SEM connectomics. *bioRxiv*, 855130 (2019).
31. Hayworth, K. J. *et al.* Gas cluster ion beam SEM for imaging of large tissue samples with 10 nm isotropic resolution. *Nat. Methods* **17**, 68-71 (2020).
32. Kizilyaprak, C., Longo, G., Daraspe, J. & Humbel, B. M. Investigation of resins suitable for the preparation of biological sample for 3-D electron microscopy. *J. Struct. Biol* **189**, 135-146 (2015).
33. Thai, T. Q. *et al.* Rapid specimen preparation to improve the throughput of electron microscopic volume imaging for three-dimensional analyses of subcellular ultrastructures with serial block-face scanning electron microscopy. *Med. Mol. Morphol.* **49**, 154-162 (2016).

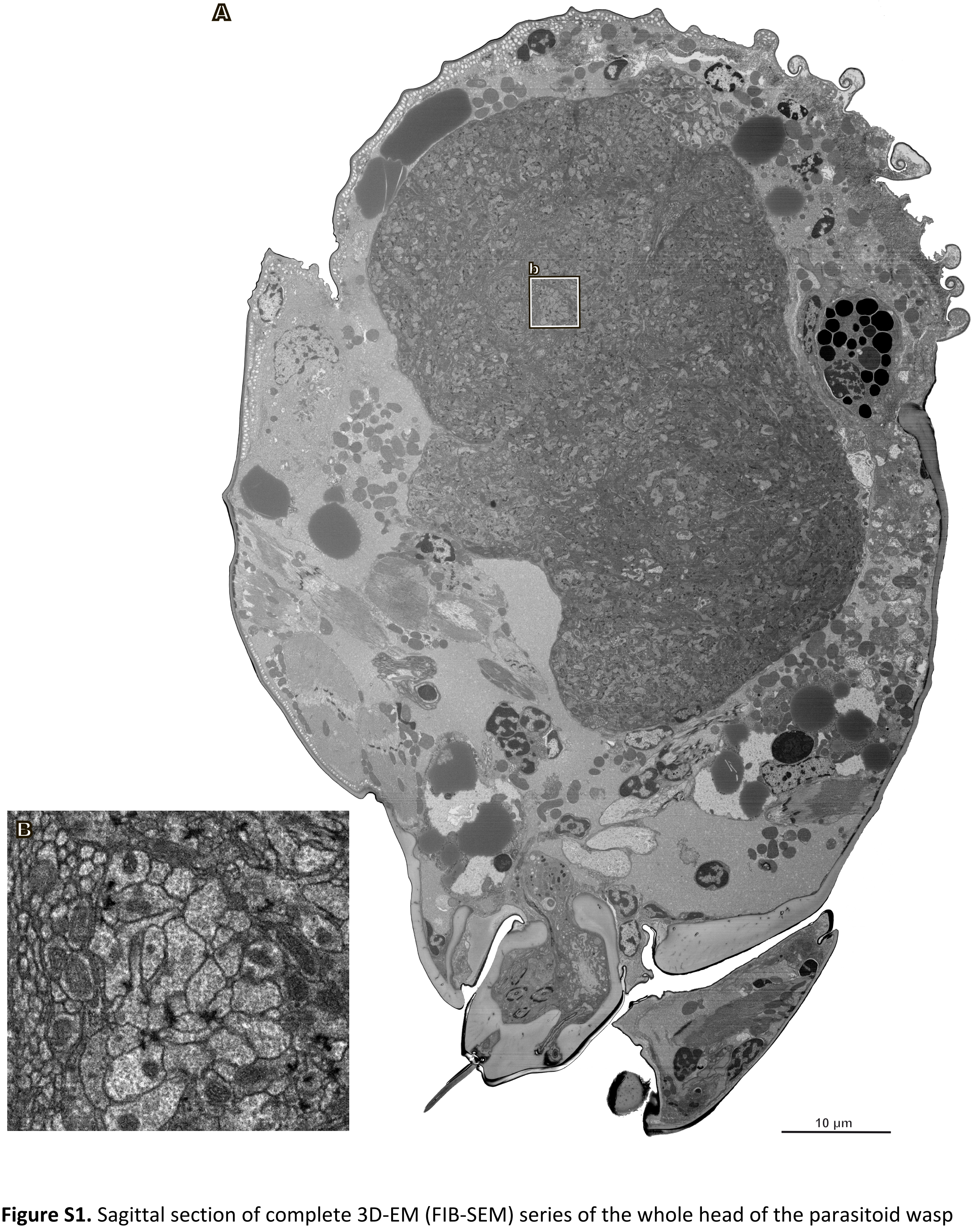

Figure S1. Sagittal section of complete 3D-EM (FIB-SEM) series of the whole head of the parasitoid wasp *Megaphragma amalphitanum* (same stack as in Fig. 3, S2). Magnified fragment of a central portion of the neuropil of the brain (B).

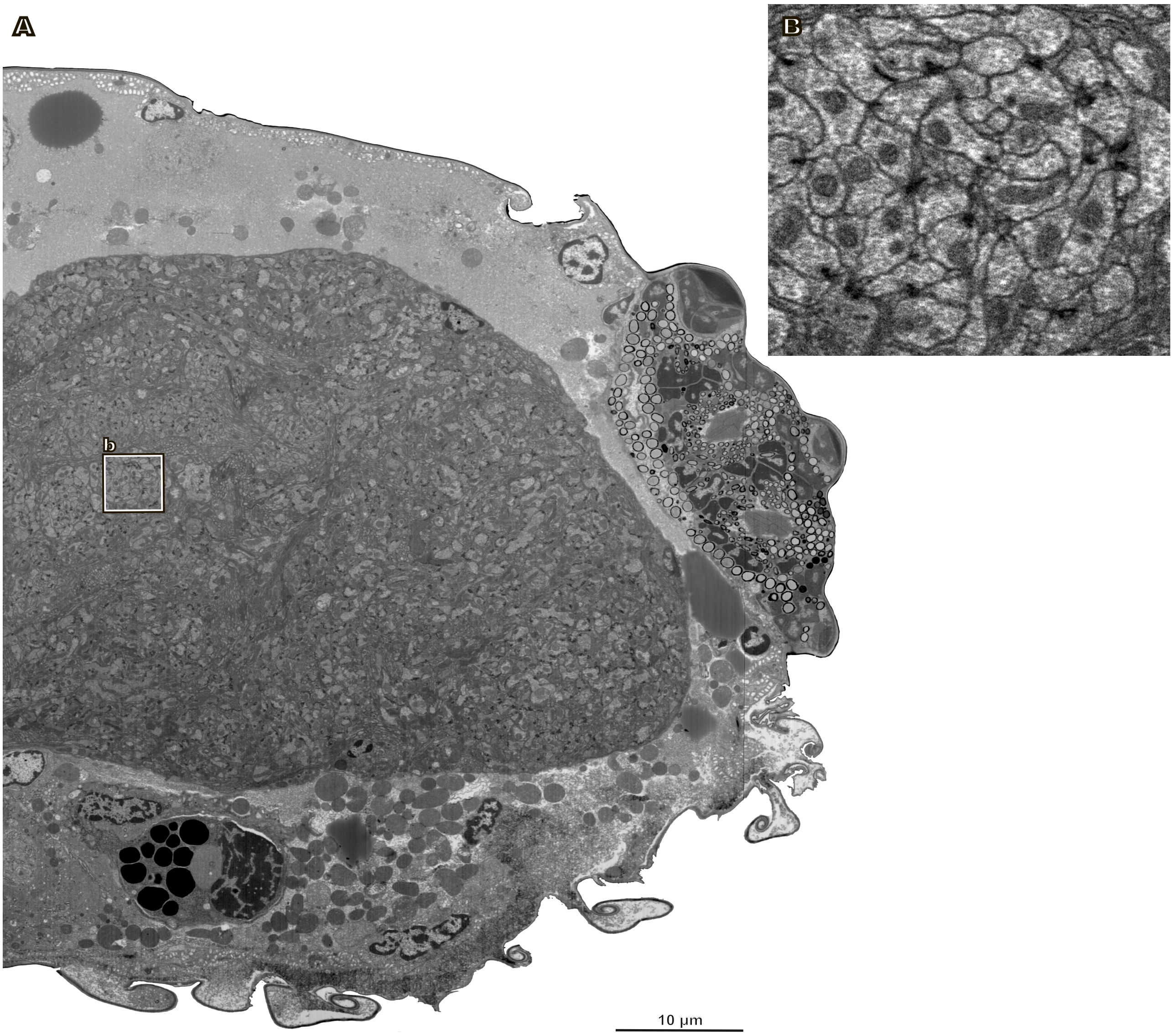

Figure S2. Frontal section of complete 3D-EM (FIB-SEM) series of the whole head of the parasitoid wasp *Megaphragma amalphitanum* (same stack as in Fig. 3, S1). Magnified fragment of a central portion of the neuropil of the brain (B).

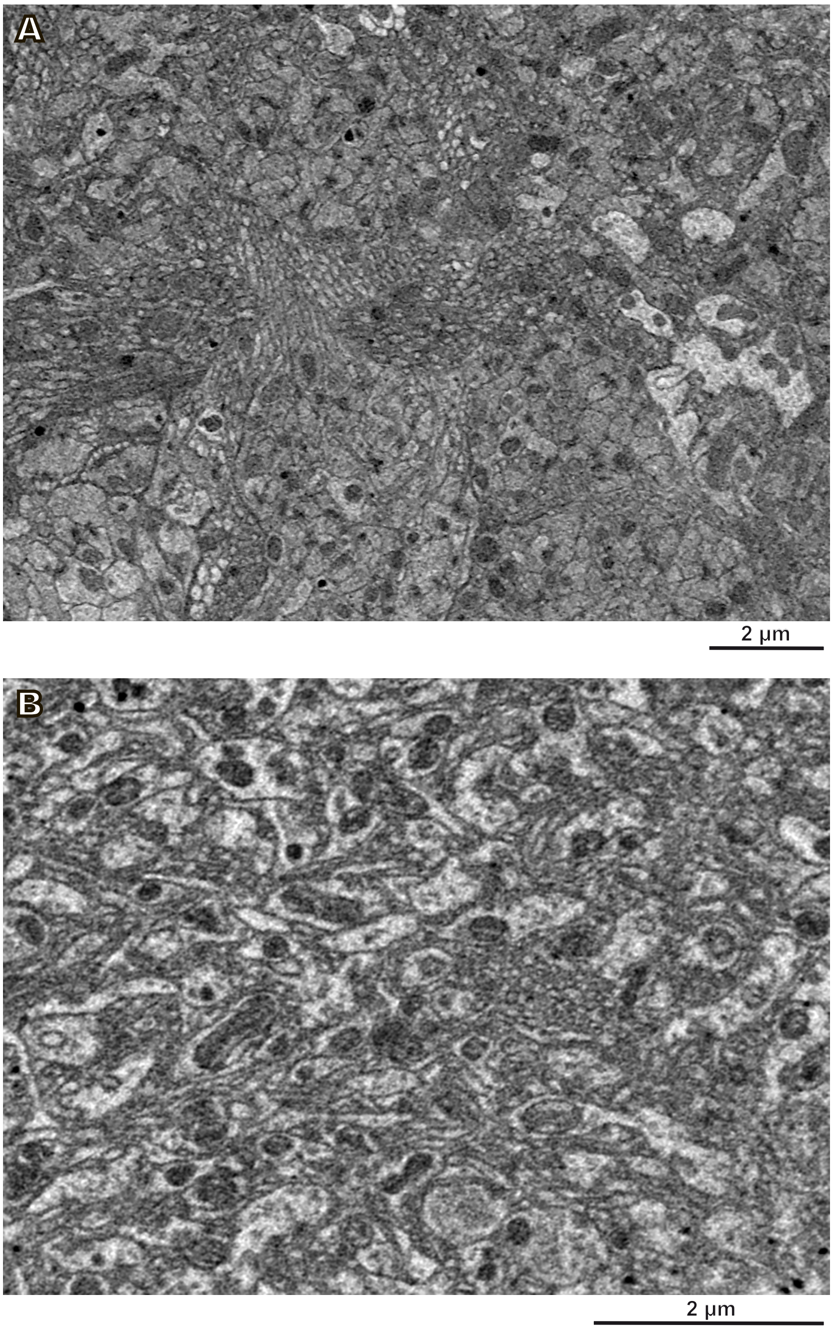

Figure S3. Examples of SBF-SEM images of central portions of the neuropil of the brain of the parasitoid wasp *Megaphragma amalphitanum*.

List of chemicals

I. Needed directly for sample preparation according to our protocol

- Acetone (Ac) (AppliChem 481007.1611) drying with molecular sieves (Sigma 208582).
- Aspartic acid (Sigma A7219). For preparing 0.03M solution, dissolve 0.199 g aspartic acid in 50 mL H_2O. Dissolving at RT using a rotator takes several hours. The solution can be stored at 4 °C for about a month.
- Double-distilled water, freshly prepared (ddH_2O).
- Epoxy embedding medium kit (Epon 812, Sigma 45359). Mix before using in proportion (V) 10 Epon + 5 DDSA + 5 MNA + 0.35 DMP-30. The mixture can be stored at −25 °C for several months, but it is preferable to use a freshly prepared mixture.
- Ethanol (EtOH) drying with molecular sieves (Sigma 208582). For dehydration, 30%, 50%, 70%, and 90% solutions have to be prepared prior to using and cooled to 4 °C
- Glutaraldehyde (GA) (EMS 16220). For preparing fixatives 1 and 2, 1% and 2% solutions (V/V) in CB have to be prepared prior to using and cooled to 4 °C
- Hydrochloric acid (HCl) (Sigma Tech 20-4). For preparing 0.2M solution, dissolve 0.84 mL HCl in 50 mL ddH_2O. The solution can be stored at 4 °C for several months.
- Lead nitrate (EMS 17900). For preparing process solution, dissolve 0.066 g lead nitrate + 10 mL 0.03M aspartic acid and pH adjusted to 5.5 with 1M KOH (about 350 µL). After preparation, keep for 30 min at 50 °C.
- Osmium tetroxide (OsO_4) (EMS 19134). For using initially crystal OsO_4, it has to be dissolved prior to using in ddH_2O at a concentration of 4–5%. Frozen aliquots of the solution at −25 °C for several months.
- Potassium ferrocyanide (hexacyanoferrate II) (FeCN) (Sigma P3289). Working solution of 1% FeCN in CB (W/V) is prepared prior to using.
- Potassium hydroxide (KOH) (AppliChem 141515.1210). For preparing 1M solution, dissolve 2 g KOH in 50 mL ddH_2O. The solution can be stored at 4 °C for several months.
- Sodium cacodylate trihydrate (Sigma 20840). For preparing 0.2M buffer (CB), dissolve 2.14 g cacodylate in 50 mL ddH_2O and have pH adjusted to 7.2 with 0.2M HCl (about 4.2 mL).
- Uranyl acetate (UA) (Serva 77870). For preparing 1% process solution (W/V), dissolve UA in fresh ddH_2O prior to using and filter, but the solution can be stored at 4 °C for several days.

II. Additional reagents used in this study

- Araldite 6005 (EMS 13920)
- Bovine serum albumin (Sigma A2058)
- Calcium chloride (EMS 12340)
- Copper sulfate (Sigma 61230)
- Dimethyl sulfoxide (EMS 13390)
- Durcupan embedding media kit (Sigma 44610)
- Formaldehyde Aqueous Solution (PFA)(EMS 15710)
- Formamide (Sigma 47670)
- Glycerol (Sigma G5516)
- Hard-Plus resin-812 (EMS 14115)
- Hexadecene (Sigma H2131)
- Hydrogen Peroxide (EMS 16790)
- Lead Citrate (EMS 17800)
- Methanol (Vekton)
- Periodic acid (EMS 19325)
- Phosphotungistic acid (EMS 19500)
- Picric Acid (EMS 19550)
- Potassium ferricyanide (Sigma 702587)
- Potassium permanganate ($KMnO_4$) (AppliChem 141527.1210)
- Propylene oxide (PO) (EMS 20412)
- Pyrogallol (Sigma 254002)
- Ruthenium red (EMS 20600)
- Silver conductive Adhesive (EMS 12686-15)
- Sodium hydroxide (NaOH) (AppliChem 141687.1211)
- Sodium citrate (EMS 21140)
- Sodium phosphate dibasic (EMS 21180)
- Sodium phosphate monobasic (EMS 21190)
- Spurr resin kit (EMS 14300)
- Sucrose (EMS 21600)
- Tannic acid (TA) (EMS 21700)
- Thiocarbohydrazide (Sigma 88535)
- Triton X-100 (EMS 22140)

List of equipment and supplies

I. Needed directly for sample preparation according to our protocol

- Micro-Needles (EMS 62091-01)
- Tweezers (Dumont 0103-5-PO)
- Concavity Slides (EMS 71878-03)
- Mechanical Pipette (Sartorius Proline Plus 728010, 728060, 728070, 728080) and tips for it.
- Iso-Freeze PCR Racks (SSI, 5640-T6)
- Workstation and 96-Place WorkUp Rack (SSI-5220-29)
- Plastic tubes (Sarstedt 72.737.002, 72.735.002, 72.690.001, 72.695.500, 60.558, 60.551, 62.559.001)
- Plastic containers (Sarstedt 75.9922.745, 75.9922.421)
- Flat embedding silicone molds (EMS 70901-CB)
- Syringe filters (Corning 431222)
- pH meter (San-Xin SX-620)
- Stirring hotplate (IKA RH B-KT/C)
- Stereoscopic dissecting microscope (Motic SMZ-171)
- Rotator (BioSan Multi Bio RS-24)
- Scale (Ohaus Explorer Pro EP 214C)
- Bidistillator (GFL 2304)
- Fume hood (HimBioTest ShV/1)
- Constant-temperature oven (SKTB SPU TC-1/20)
- Refrigerator (Samsung RL-63 GCBMG)
- Individual protection means (nitrile gloves, lab coat, protective glasses)

II. Additional equipment used in this study

- Compound microscope Olympus BX43
- Ultramicrotome (Leica UC6).
- Microtome (Leica RM 2255)
- Diamond Knifes (DiATOME Ultra and Histo Jumbo).
- Transmission electron microscopes Jeol JEM-1011 and JEM-1400.
- DualBeam Focused Ion Beam-Scanning Electron Microscopes (FIB-SEM) FEI Quanta FEG, FEI Helios, modified combined Zeiss Merlin SEM and Capella FIB (Janelia, Hess Lab)
- X-ray micro-CT (Xradia Versa 3D XRM-510)
- Serial block-face scanning electron microscope (SBF-SEM) FEI Teneo.
- Microwave processor (Panasonic NN-SD372S microwave oven modified for sample processing: even distribution of microwaves, inverter control of power, cooling, control of temperature)
- Blades (Tissue-Tek 4689)
- Specimen Mounts for Scanning Electron Microscopes (EMS 75200)
- Grid Storage Box (EMS 71150)
- Microscope slides and cover glasses (Thermo Scientific J2800AMNZ, 3322)
- TEM grids (EMS FFGA1000-Cu, FFGA600-Cu, FCFGA1000-Cu, FCFGA600-Cu)
- High-pressure freezer (Leica EM HPF-100)
- Automatic freeze substitution system (Leica EM ASF2)
- Cryo tubes (EMS 34506)
- Freezer hats (EMS 71167)
- Hot plate (Microstat 30/80)
